# Can immunotherapy be useful as a “functional cure” for infection with Human Immunodeficiency Virus-1?

**DOI:** 10.1186/1742-4690-9-72

**Published:** 2012-09-07

**Authors:** Guido Vanham, Ellen Van Gulck

**Affiliations:** 1 Virology Unit, Department of Biomedical Sciences, Institute of Tropical Medicine of Antwerp, Nationalestraat 155, B-2000, Antwerpen, Belgium; 2 Department of Biomedical Sciences, University of Antwerpen, Antwerpen, Belgium; 3 Present address: Community of Research Excellence and Advanced Technology (C.R.E.A.Te), Division of Janssen, Beerse, Belgium

**Keywords:** HIV, SIV, Immune therapy, Functional cure, Therapeutic vaccine, CD8 T cells, Macaque trials, Human trials

## Abstract

Immunotherapy aims to assist the natural immune system in achieving control over viral infection. Various immunotherapy formats have been evaluated in either therapy-naive or therapy-experienced HIV-infected patients over the last 20 years. These formats included non-antigen specific strategies such as cytokines that stimulate immunity or suppress the viral replication, as well as antibodies that block negative regulatory pathways. A number of HIV-specific therapeutic vaccinations have also been proposed, using *in vivo* injection of inactivated virus, plasmid DNA encoding HIV antigens, or recombinant viral vectors containing HIV genes. A specific format of therapeutic vaccines consists of *ex vivo* loading of autologous dendritic cells with one of the above mentioned antigenic formats or mRNA encoding HIV antigens.

This review provides an extensive overview of the background and rationale of these different therapeutic attempts and discusses the results of trials in the SIV macaque model and in patients. To date success has been limited, which could be explained by insufficient quality or strength of the induced immune responses, incomplete coverage of HIV variability and/or inappropriate immune activation, with ensuing increased susceptibility of target cells.

Future attempts at therapeutic vaccination should ideally be performed under the protection of highly active antiretroviral drugs in patients with a recovered immune system. Risks for immune escape should be limited by a better coverage of the HIV variability, using either conserved or mosaic sequences. Appropriate molecular adjuvants should be included to enhance the quality and strength of the responses, without inducing inappropriate immune activation. Finally, to achieve a long-lasting effect on viral control (i.e. a “functional cure”) it is likely that these immune interventions should be combined with anti-latency drugs and/or gene therapy.

## Review

### Introduction: functional cure and natural immune control of HIV

During last year’s International AIDS Society (IAS) Conference (July 2011) the “Rome Statement for an HIV Cure” was issued, pleading for “the development of a functional cure which, without completely eliminating the virus from the body, would permanently suppress its replication and considerably diminish viral reservoirs, possibly leading to the long-term remission of patients, in the absence of antiretroviral drugs” (see http://www.iasociety.org/Default.aspx?pageId=584). More recently (July 2012), the dedicated IAS Working Group organized a well-attended symposium in Washington “Towards an HIV Cure”, accompanied by a “Perspective” paper in Nature Reviews Immunology. Two possibilities for cure were distinguished: “first, the elimination of all HIV-infected cells (a sterilizing cure); and second, the generation of effective host immunity to HIV that would result in lifelong control of the virus in the absence of therapy, despite not achieving the complete eradication of HIV (a functional cure)” [1]. The prototypical example of an HIV cure is the well-known “Berlin patient”, who, after elimination of his own immune system by irradiation in the context of acute myeloid leukemia treatment, was transplanted with hematopoietic stem cells (HSC) from a homozygous CCR5 delta32 donor and subsequently remained virus-free without any antiretroviral treatment for over 5 years at the time of writing [2]. Nevertheless, during the recent symposium, data were presented on some remaining detectable HIV DNA in rectal biopsies, despite having plasma RNA levels below 1 copy and a complete absence of viral DNA, RNA or cultivable virus from peripheral blood mononuclear cells (PBMC). Hence, this much-acclaimed patient is probably an example of a “functional” rather than of a “sterilizing” cure (S Palmer personal communication). As a consequence of this unique success story, several research groups are now attempting to knock out the CCR5 genes in various cell types, including HSC [3]. Results of syngeneic CCR5(−) HSC transfer in humanized mice indicate that, upon infection, HIV-1 viral levels are clearly lower, but not absent [4]. Since it remains uncertain whether this cumbersome genetic therapy will offer a cure for all patients [5], other strategies need to be considered, including immunotherapy and anti-latency drugs, as proposed by the Working Group (http://www.iasociety.org/Default.aspx?pageId=606).

### Role of HIV-specific immunity in viral control

Several excellent reviews on immune mechanisms of HIV control have recently been published [6-8]. In the earliest phase of infection innate responses, including type-1 interferon (IFN-α/β) and Natural Killer (NK) cells partly control virus replication. Only after a few weeks do HIV-specific CD4 and CD8 T cell responses as well as antibody responses emerge and reduce viral load (VL) to a patient-specific setpoint [9]. For a long time, interleukin-12 (IL-12) has been thought to have an essential immune-regulatory role in the induction of a “Th1-skewed cellular response”, characterized by an optimal interplay between IL-2/IFN-γ producing CD4 helper and CD8 effector T cells, which is crucial for the adaptive phase on anti-viral responses [10].

Examples of the “naturally occurring” functional cure in HIV infection include the so-called “elite controller” (EC) HIV-1 patients, who are therapy-naïve but nevertheless keep their “viral setpoint” below 50 RNA copies per ml plasma [11]. In addition, rare non-controllers acquire a “secondary” controller status after prolonged “Highly Activate Antiretroviral Therapy” (HAART), initiated either in the acute phase [12,13] or, even more rarely, in the chronic phase [14,15].Clearly, this uncommon phenomenon of “post treatment control” (PTC) is seemingly more easily induced if HAART is started in the acute as opposed to the chronic phase, pointing to a contribution of a less damaged immune system and/or a lower proviral reservoir.

Whereas the in-depth study of PTC is just beginning, more data are available on EC. Decreased viral replication capacity and genetic host factors have been identified in a percentage of EC. Unfortunately, these factors are largely impossible to operationalize with the present state-of-the art technology. However, an association has been found between viral control and HIV Gag-specific CD4 and CD8 T cell responses, but not Env-specific T cells or Env-specific neutralizing antibodies, pointing to an important role for HIV-specific T cell immunity towards more conserved structural parts of the virus such as Gag [16,17].

Effective CD8 T cells, capable of keeping the plasma viral load under control, are characterized by a central and effector-memory phenotype, low levels of aberrant activation and exhaustion markers (e.g. CD38 and programmed death (PD)-1), and preserved costimulatory receptors (e.g. CD28). Effective HIV-specific CD8 T cells produce multiple cytokines and effector molecules; they have preserved proliferative capacity; and their T cell receptors show high avidity and/or cross-reactivity, preferentially recognizing conserved epitopes in Gag, thus leaving little opportunity for immune escape [16,17]. Importantly, these CD8 T cells also show a high-avidity cytolytic potential against infected cells [18] and have the capacity to suppress viral replication *in vitro*[19,20]. These observations provide a rationale to attempt to develop strategies to enhance immune control by “therapeutic vaccination” [11,21].

In order to induce and maintain this type of effective CD8 T cells, a well-coordinated interaction with dendritic cells (DC) and CD4 T cells is important [22]. HIV-specific CD4 T cells play an important role by producing “helper” cytokines, such as those triggering the “common γ chain receptors” (IL-2, IL-7 and IL-21), as well as by upregulating costimulatory membrane markers such as CD40L as well as CD80 and CD86, which promote CD8 T cell survival, proliferation, cytotoxicity and virus-suppressive capacity [6,23]. DC can directly activate CD8 T cells, but also induce effective CD4 T cell help to CD8 T cells. Therefore, harnessing the DC function to improve the quality of T cell responses against HIV can be an important mechanism in immunotherapy [24,25].

### The danger of an activated immune system

Progressive HIV infection is characterized by persistently increased levels of various soluble inflammatory markers, dysregulation of T cell surface markers, and upregulation of receptors for immune suppressor signals such as PD-1 and cytolytic T-lymphocyte antigen (CTLA)-4. This inappropriate immune activation is partly due to a persistent VL, but in addition endotoxins originating from microbial translocation through a “leaky gut” and “hypersensitivity” to type 1 IFN have been implicated (for review see [26]). A sizeable body of evidence suggests that this persistent “immune activation syndrome” constitutes a bad prognostic sign, independent from CD4 T cell count, plasma VL and cellular proviral load, even under suppressive HAART [27]. Unsurprisingly, inappropriate T cell activation is associated with increased susceptibility of CD4 T cells to infection and decreased T cell responsiveness to antigenic stimulation, including reduced IL-2 production, and increased apoptosis. This activation-induced “T cell exhaustion” conceptually limits the possibilities of immunotherapy and therefore it is important to provide sufficient co-stimulatory signals [28], without increasing susceptibility of target cells to HIV.

### The role of HAART and viral reservoirs

HAART reduces VL and immune activation, and therefore it was hoped that long-term HAART would allow the immune system to recover its capacity to control the virus. A number of “structured treatment interruption” (STI) trials have indicated that while immune responses to HIV were boosted, the VL rebounded to pre-treatment levels in most patients (except for the rare “secondary controllers” or PTC), suggesting that infectious virus is not a good antigen for immune therapy [29]. The fundamental problem to fully eradicate the virus is the persistence of a “latent” reservoir. Neither long-term treatment with classical HAART cocktails (based on reverse transcriptase and protease inhibitors) nor treatment intensification with newer integrase or entry-inhibitors can consistently reduce this reservoir (for review see [30]).

One proposed strategy for cure is to activate the latent provirus under coverage of HAART: the rescued virus will kill the producer cell, but cannot infect new targets . Many excellent reviews have been dedicated to this complicated challenge [3,31,32]. The y-chain cytokine interleukin-7 (discussed below for its immune stimulating potential) is currently under study for its potential to “purge” the latent HIV reservoir (ERAMUNE trial, http://www.clinicaltrials.gov). A number of pharmacological agents, including (combinations of) histone-deacytelase inhibitors, NF-κβ activating agents and others have shown some HIV-rescuing activity *in vitro.* This has been accompanied, however, by global T cell activation and, until now, no convincing favorable clinical data have been reported [30]. Many pharmaceutical companies are currently screening compound libraries to find novel factors that could more potently and more selectively rescue the latent virus, but this topic is beyond the scope of the present review.

In the context of immunotherapy, however, anti-latency drugs are relevant; even if they were unable to “purge” the reservoir by themselves. In fact, complete latency renders infected cells invisible to the immune system, precluding targeting by therapeutic vaccination. Anti-latency drugs could overcome this hurdle, because they induce expression of viral proteins that would mark the infected cells as targets for immune elimination [33,34].

In conclusion, several principles can be proposed for immunotherapy. These include a non-specific enhancement of anti-viral immune responses by various immune stimulators, including type-1 IFN, IL-12 and the so-called common γ-chain signaling cytokines, related to IL-2. In addition, blocking antibodies against immune suppressive receptors such as PD-1 and CTLA-4 could also provide beneficial immune stimulation. On the other hand, a range of antigenic formats have been proposed to induce HIV-specific T cell responses, in order to elicit more effective CD8 T cell-mediated immune surveillance.

In the following paragraphs, we will explain the rationale of each strategy and then focus mainly on therapeutic vaccination trials in Simian Immunodeficiency Virus (SIV)-infected macaques and HIV infected patients, critically investigating their potential to complement (and ultimately replace) anti-retroviral drug therapy. Clearly, no definite strategy for a cure has been established yet, but encouraging results are emerging and the concepts are slowly but surely maturing.

### Non-antigen specific immune therapies

An overview is presented in Table1.

**Table 1:** Non antigen-specific immune therapy

**Name Trial**	**Species**	**Baseline patients characteristics**	**Type of immune therapy**	**Route and frequency of administration**	**Number of patients**	**Outcome**	**References**
Phase I dose escalation trial	Human	HAART	IL-12	sc, single dose	47	Dose related increase in serum IFN-γ levels, NK and CD8 T cell numbers.	[35]
Phase I randomized placebo controlled	Human	HAART	IL-12	sc, multi dose twice weekly for 4 weeks.	56	Well tolerated at doses up to 100 ng/kg. Dose related increase in neopterin levels. No differences in other immunological parameters or viral load	[36]
Three randomized trials	Human	Mono- or dual treated	IL-2	iv intermittent 5 days regimen	155	Higher CD4 T cell count, lower VL and 43% reduction in risk of disease progression or death.	[37]
SILCAAT	Human	HAART Low CD4 T cell count	IL-2	sc 6 cycles of IL-2 twice daily for 5 consecutive days	1695	Sustained effect on CD4 T cells without affecting clinical progression	[38]
ESPRIT	Human	HAART High CD4 T cell count	IL-2	sc 3 cycles of IL-2 twice daily for 5 consecutive days	4111	Temporary effect on CD4 T cells without affecting clinical progression.	[38]
ANRS 119 randomized clinical trial	Human	Therapy naïve	IL-2	sc 3 cycles of IL2 for 5 consecutive days	130	Sustained increase in CD4 T cells without affecting viral load.	[39]
ANRS-NIH ILIADE	Human	HAART with high CD4	IL-2	sc 3 cycles of IL2 for 5 consecutive days. ATI on week 24	148	Delay HAART resumption following treatment interruption. No effect on viral load.	[40]
Random placebo controlled trial	Human	Therapy naïve	IL-7	sc, single injection	25	Increased numbers of circulating CD4 and CD8 T cells. Transient increase in VL.	[41]
EudraCT (open label phase I/IIa)	Human	HAART	IL-7	sc repeated injections. Eight doses 3 times a week	13	Expansion of naïve CD4 and CD8 T cells.	[42]
	Rhesus macaque	Chronic SIV	IL-7	sc 4 injections every 3 weeks	9	Counteracts IFN-α induced lymphopenia. Increasing circulating CD4 T cells.	[43]
	Indian rhesus macaque	Chronic SIV + HAART	IL-15	sc twice a week from day 0-day42	16	Delayed viral suppression. Failed to enhance antigen-specific CD4 T cell reconstitution at mucosal and lymphoid sites. Upon ATI loss of CD4 T cells more rapidly.	[44]
	Indian rhesus macaque	Chronic SIV	IL-21	2 iv injections 7 days apart and 3 sc doses 23 days after 2^nd^ vaccination.	7	Safe and well tolerated. Increased cytotoxic potential of T cells, increased SIV antibody production.	[45]
	Indian rhesus macaque	Chronic SIV	Blocking Ab to PD-1	Iv	14	Expansion virus specific CD8 T cells and B cell activation. Reduction in plasma VL and prolonged survival.	[46]
	Rhesus macaque	Chronic SIV + HAART	Blocking Ab to CTLA4	iv	16	Increase CD4 and CD8 T cell responses and drop of viral RNA.	[47]
	Indian rhesus macaque	Chronic SIV + HAART	Blocking Ab to CTLA4	iv	10	No expansion SIV specific T-cells. Increased activation of T cells and increased viral replication at mucosal sites.	[48]

#### Cytokine therapies

##### Type-1 interferon

During the acute phase of HIV infection, high levels of serum IFN-α are part of the innate antiviral response, as *in vitro* results showed that IFN-α indeed reduced HIV replication in both CD4 T cells and monocytes-macrophages. However, the role of type-1 IFN in HIV pathogenesis is rather ambiguous, since in the chronic phase, serum markers of increased IFN activity, such as neopterin and β2-microglobulin have consistently been associated with a bad prognosis [49].

Based on the first premise (type 1 interferon = anti-viral), *in vivo* IFN-α treatment was attempted in the era before anti-retroviral drugs were available, but failed to provide benefit in Acquired Immune Deficiency Syndrome (AIDS) patients; whereas in infected patients with preserved immunity a trend to a better clinical outcome was noted. Later, IFN-α was combined with monotherapy or bi-therapy of nucleoside reverse transcriptase inhibitors (NRTI). A trend for increased antiviral effects was noted, but this benefit was offset by rather serious flu-like side effects [50]. Once efficient HAART tri-therapy became available, combinations with type 1 interferons were abandoned for the indication of HIV infection alone (though they are still in use for selected cases of chronic hepatitis and HIV co-infection).

Since elevated IFN-α levels are suspected to play a role in pathological immune activation, Zagury et al. attempted to immunize HIV patients against this cytokine. The subgroup of patients with a rise in anti-IFN-α antibodies had a significantly lower incidence of HIV-1-related events compared with placebo recipients and vaccinees who failed to develop antibodies [51].

A different approach was used more recently with the anti-malarial drug chloroquine. Preliminary evidence *in vitro* and *in vivo* indicated that chloroquine reduces IFN-α production and decreases the level of immune activation [52]. A randomized double blind placebo-controlled trial in therapy-naïve patients, however, failed to show any favorable effect on immune activation and, unfortunately, did result in a greater decline in CD4 T cell count and increased VL [53].

##### Interleukin-12

A Th1/Th2 imbalance has traditionally been regarded as a hallmark of HIV-related immune dysfunction [54]. IL-12 represents the archetypical Th1 switching agent: it induces type II interferon (IFN-γ) production by T cells and NK cells and increases their cytotoxic capacity against virally infected cells [10]. A number of *in vitro* studies in PBMC cultures from HIV infected subjects confirmed that IL-12 increased Th1 responses [55,56]. Before the HAART era, two phase 1 studies of subcutaneous (sc) IL-12 were conducted in medically stable HIV-infected patients. Single doses of sc IL-12 between 30 and 300 ng/kg were reasonably tolerated and induced a dose-related increase of serum IFN-γ, but failed to influence CD4 T counts or VL [35]. In a subsequent placebo-controlled multi-dose trial, IL-12 was tolerated in doses up to 100 ng/kg, but again no effect on CD4 T counts or VL was observed [36]. Apparently, repeated IL-12 administration resulted in “tolerance”, and overdosing could even result in paradoxical immune suppression through activation of NO production [57,58]. Nowadays, in many experimental immunization schedules with HIV antigens in either plasmid DNA or viral vectors, an expression cassette for IL-12 is being added, thus focusing IL-12 expression at the site of immunization and avoiding systemic side effects.

##### Common γ chain signaling cytokines

These cytokines include IL-2, -4, -7, -9, -13, -15 and -21 and bind to receptor complexes that include the so called common γ signaling chain. Among these, IL-4, -9 and -13 skew the immune system towards Th2 responses, considered pathogenic in HIV infection. The other γ chain cytokines have potentially beneficial effects to overcome immune dysfunction [59,60].

IL-2 enhances both CD4 T cell proliferative and CD8 T cytolytic functions, but may also induce peripheral tolerance by activating regulatory T cells (Treg) [61]. Deficient IL-2 production upon antigenic stimulation has consistently been reported as a hallmark of HIV-related immune dysfunction, from the early 80’s on [62,63]. Interleukin-7 has been shown to play a crucial role in promoting expansion and maintenance of T cells. IL-7 production is increased during HIV-induced lymphopenia, but this feedback is apparently not sufficient to maintain T cell homeostasis [64]. The primary role of IL-15 is to expand the effector-memory subset of CD8 T cells, which is crucial in immune control, and to promote survival of NK cells. Several studies have shown that IL-15 production is compromised in AIDS patients, and supplementation of IL-15 improves the function of immune cells from these patients *in vitro*[65]. Finally, IL-21 promotes proliferation and accumulation of antigen-specific CD8 effector T cells, increases their survival and cytolytic potential, especially in synergy with other cytokines, and promotes differentiation of naive CD4 T cells without inducing Treg. IL-21 production is compromised early on during HIV infection and it is only partly restored by HAART [66].

##### Therapeutic use of interleukin-2

A number of smaller studies in the 90’s evaluated systemic IL-2 therapy in HIV patients treated with mono- or dual- NRTI drug therapy. A pooled analysis suggested that this type of immunotherapy resulted in higher CD4 T counts, lower VL, as well as fewer opportunistic infections and deaths [37]. In order to confirm the observed effects in fully treated patients, two multicenter placebo controlled trials with IL-2 were initiated. In both the SILCAAT and the ESPRIT trial, “induction” and “maintenance” cycles of sc IL-2 were added to virus-suppressive HAART. In both trials a positive effect on peripheral CD4 T counts was confirmed (temporary in ESPRIT and sustained in SILCAAT). VL was continuously suppressed (by HAART), but the occurrence of opportunistic infections or death was not altered as compared to HAART only [38]. Two smaller ANRS (Agence de Recherche sur le SIDA) studies addressed additional questions. ANRS 119 showed that intermittent IL-2 therapy in HAART-naïve patients with CD4 T counts between 300–500 cells/mm^3^ could induce a significant and sustained increase in CD4 T cells, though unfortunately without affecting VL, but, based on CD4 T criteria, initiation of HAART could be deferred [39]. The ANRS-NIH ILIADE trial suggested that administration of 3 cycles of IL-2 in HIV-infected patients with high CD4 T cell counts and controlled VL on HAART, could allow a significant delay in HAART resumption following treatment interruption [40]. Taken together, the potential beneficial effects of systemic IL-2 are rather limited; additionally, it is not devoid of side effects, and sc administration is cumbersome. Therefore, adjunctive systemic IL-2 therapy has not been adopted in routine clinical care. This negative appreciation, however, does not preclude a local targeted IL-2 administration in conjunction with therapeutic vaccination.

##### Therapeutic use of interleukin 7

A single injection of IL-7 in untreated HIV-infected subjects increased the number of circulating CD4 and CD8 T cells, mainly those of the central-memory type, without affecting the frequency of Tregs, but with a slight increase of viral RNA (blip) in half of the patients [41,42]. A prospective open label trial (EudraCT) investigated the effect of repeated sc IL-7 administration in those patients who remained lymphopenic under fully virus-suppressive HAART. A significant expansion of circulating memory, but also naive, CD4 and CD8 T cells was observed, without tolerability problems and without increases in T cell activation or proviral load. Moreover, IL-7 treatment counteracted IFN-α therapy-induced lymphopenia and stimulated SIV-specific cytotoxic T lymphocyte responses in SIV-infected rhesus macaques [43]. Ongoing phase 3 trials should indicate whether IL-7 has a role in correcting lymphopenia in patients who fail to recover CD4 T cell counts under HAART (or in purging the viral reservoir, as discussed above).

##### Therapeutic use of interleukin 15

Interleukin-15 has several theoretical advantages over IL-2, in that it inhibits apoptosis and enhances expansion of both CD4 and CD8 memory T cells, as well as NK cells [67]. However, systemic IL-15 administration in chronically SIV infected macaques treated with HAART resulted in a delay in viral suppression; and, when HAART was interrupted, IL-15 co-treated animals experienced a more rapid loss of CD4 T cells as compared to HAART-only treated animals [44]. These negative results have discouraged further systemic administration, though IL-15 might still be useful in conjunction with a HIV vaccine [68].

##### Therapeutic use of interleukin 21

Interleukin-21 might be the most suitable γ-chain cytokine for immunotherapeutic purposes. IL-21 by itself induced potent antiviral activity in human CD8 T cells [69] and augmented the lytic potential of NK and CD8 T cells from HIV-infected subjects [70-72]. HIV-1 specific IL-21 producing CD4 T cell responses also contributed to durable viral control through the modulation of HIV-specific CD8 T cell function [73]. The *in vivo* relevance of all these *in vitro/ex vivo* observations was most recently confirmed in a small trial of systemic IL-21 administration in chronically untreated SIV infected rhesus macaques. IL-21 administration was well tolerated, augmented the cytotoxic potential of both T cells and NK cells, and promoted B cell differentiation with increased SIV antibody production, without an increase in cellular activation or plasma VL [45]. These results encourage further testing of IL-21 in conjunction with HAART and/or a specific therapeutic vaccine.

##### Blocking negative regulatory receptors

In an elegant *in vitro* study, Shankar et al. recently showed that co-culture of HIV-infected DC with naïve T cells induced elevated membrane expression of a broad array of negative costimulatory molecules such as PD-1 and CTLA-4, with a concomitant decreased expression of the effector cytokines [74]. Many of these characteristics of T cell anergy and exhaustion have also been described in T cells from HIV-infected subjects.

High levels of PD-1 were shown on both CD4 and CD8 T cells but particularly on HIV-1 specific cytolytic T cells (CTL), and were correlated with CTL dysfunction and apoptosis. PD-1 expression correlated directly with VL and disease progression and inversely with CD4 T counts. The natural ligand PD-L1 was significantly upregulated on antigen-presenting cells (APC) from HIV-infected individuals. Importantly, antibody-mediated blocking of this interaction improved HIV-specific T cell functions *in vitro*[75,76].

CTLA-4 is overexpressed on CD4 T but not on CD8 T cells and more particularly on HIV-specific T cells in all infected subjects, except elite controllers. *In vitro* blocking of CTLA-4 augmented HIV-specific CD4 T cell proliferation. Additionally, CTLA-4 signaling resulted in high CCR5 expression and enhanced susceptibility to viral infection [75].

##### Contrasting effects of therapeutic blocking of PD-1 and CTLA-4

Administration of blocking antibodies to PD-1 during chronic SIV infection in macaques had remarkably positive effects. A rapid expansion of virus-specific CD8 T cells with improved functional quality, as well as B cell activation with increased SIV-specific antibodies was observed. These immune phenomena were associated with significantly reduced plasma VL and prolonged survival [46]. Interestingly, PD1 blocking actually reduced immune hyper-activation, expression of type 1 IFN stimulated genes (ISG) and bacterial translocation, and enhanced immunity to gut-associated pathogens [77].

The results of CTLA-4 blocking in this primate model were unfortunately less encouraging. Whereas an increase in CD4 and CD8 T responses and a decrease of viral RNA in lymph nodes were noted in an early study [47], the second trial showed no expansion of SIV-specific CTL, but an increased activation of CD4 T cells and viral replication at mucosal sites [48]. These discrepant results may point to the delicate balance of enhancing beneficial HIV-specific responses and increasing deleterious immune activation.

Clearly, blocking various individual and combined negative receptors should be studied in more detail in SIV-infected animals; additionally, the potential synergy of receptor blocking with therapeutic vaccines and other anti-retroviral therapies should be investigated.

### HIV-antigen specific immune therapies

**Figure 1:**
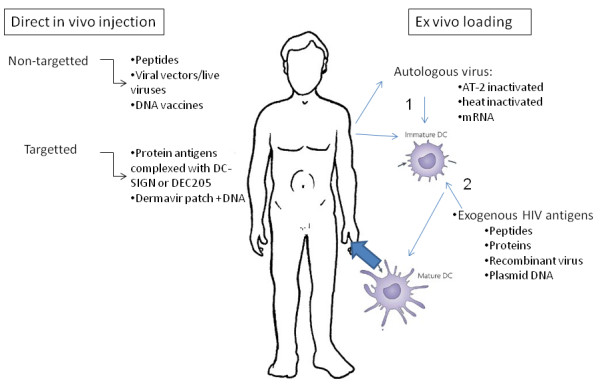
Schematic overview of HIV-antigen specific therapeutic vaccination strategies.

A general overview is presented in Figure1 and Table2.

**Table 2:** HIV-antigen specific therapeutic vaccinations

**a) Non dendritic cell-based**							
**Name Trial**	**Species**	**Baseline patients characteristics**	**Type of immune therapy**	**Route and frequency of administration**	**Number of patients**	**Outcome**	**References**
Uncontrolled clinical trial	Human	Therapy naïve	Remune®	iv	2527	Increased HIV-specific T cell responses. Positive impact on controlling the virus.	[78]
P2101B (thai open label trial)	Human	Therapy naïve	Remune®	iv every 12 weeks for 132 weeks	223	Increasing CD4 and CD8 T cell counts and stable viral load.	[79]
Randomized double blind placebo controlled trial	Human	HAART	ALVAC, ALVAC + Remune, placebo	iv injections Alvac @w 8, 12, 16, 20. Remune@w 0, 4, 12, 20	79	Safe and immunogenic. No difference between the three groups after STI on viral rebound.	[80]
Placebo controlled	Indian rhesus macaque	Chronic SIV HAART	SIV mac239 gag and env DNA vaccine	im 3 injections	23	Increase in HIV specific cellular responses and lower viral rebound in vaccinated animals.	[81]
	Indian rhesus macaque	Chronic SIV HAART	SIV mac239 gag and env DNA vaccine + IL-15	In vivo electroporation	3	Sustained polyfunctional T cells. One log decrease in VL.	[82]
	Human	HAART	DNA vaccine consisting of CTL epitopes	im injections w 0, 4, 8, 16	41	Safe and tolerable. In some persons weak responses. Overall no differences with placebo group.	[83]
VRC HIV DNA 009-00-VP double blind placebo controlled	Human	HAART	plasmid DNA encoding subtype B Gag-Pol-Nef and multiclade env	im injections w 0, 4, 8, 24	20	Poorly immunogenic. No effect on viral rebound after ATI.	[84]
	Human	HAART	Plasmid DNA multiclade	Patch	12	Broader and higher HIV specific T-cell responses. No effect on viral rebound after ATI.	[85]
ANRS 094 single arm open	Human		ALVAC vCP1433	im injections w 0, 4, 8, 12	50	Safe and immunogenic. Delay in treatment resumption of ATI.	[86]
ANRS 093	human	HAART	ALVAC + lipo 6T 4 injections followed by 3 cycles sc IL2	im injections of ALVAC w 0, 4, 8, 12. IL-2@w 16, 24, 32	71	Lower viral set point after ATI, correlated with HIV specific CD4 T cell responses.	[87]
ACTG (A5024) randomized partially blinded phase II	Human	HAART	ALVAC ALVAC + sc IL2 sc IL2 alone placebo	im injections of ALVAC w 0, 4, 8, 12. IL-2 sc for5 days in 8 week cycles	19	Lower viral rebound after ATI in ALVAC vaccines. IL-2 + vaccine boosted CD4 T cell count but had no influence on VL.	[88]
Double blind placebo controlled	Human	HAART (acute phase)	ALVAC ALVAC + Remune	iv injection of ALVAC w8, 12, 16, 20. remune w 0, 4, 12, 20	79	No influence on VL after ATI. Increased CD4 and CD8 T cell responses	[80]
CTN173 randomized controlled	Human	HAART	ALVAC, ALVAC + remune, placebo	im injections of ALVAC w8, 12, 16, 20. Remune w0, 12, 20. ATI w24	52	No lower viral setpoint. Tendency towards delay of rebound	[89]
ORVACS	Human	HAART	ALVAC vcp1420 placebo	im injections w 0, 4, 8, 20	65	Higher viral rebound in vaccines	[90]
	Human	HAART	Fowlpox gag/pol (PC)	3 im injections (w 0,4, 12). ATI w20	35	Lower man viral rebound in FC group and this is associated with IgG2 antibodies to HIVp24	[91][92]
			Fowlpox gag/pol IFN-y (FC				
			placebo				
	Indian rhesus macaque	Chronic SIV HAART	MVA gag pol and MVA env	im injections w10, 16. STI w20	18	Tendency lower viral rebound after ATI but not significant.	[93]
	Rhesus macaque	Chronic SIV HAART	MVA + Ad5 gag and env	im injection of Ad5/35 on day 74 and MVA-SIV on day 134		Vaccinated animals had higher CD4 T cell counts, SIV-specific cell-mediated immunity and anti-SIV-neutralizing antibodies. After ATI there was a sustained reduction in VL and increased CD4 T cell responses.	[94]
	Human	HAART	MVA nef	sc injections w 0, 4, 16. ATI w 18	14	Well tolerated. Induction HIV specific responses. Lower viral rebound.	[95]
	Human	HAART	MVA clade A p24/17 + CD8 T cell epitopes	id 4 week interval	18	Amplification and broadening of CD8 and CD4 T cell responses. Induction of CD8 T cell responses with capacity to inhibit viral replication *in vitro*	[96][97]
	Indian rhesus macaque	Chronic SIV HAART	Ad5 and Ad35 SIV gag, env, nef + IL15	im injections	15	Increased T cell responses no effect on viral rebound.	[98]
				w 16, 22, 36, 42			
	Human	HAART	rAd5 gag	im injections	114	Safe and well tolerated. 0.5 log lower VL 16 weeks after A-STI	[99]
				w 0, 4, 26.			
				ATI w 38 to 54			
**b) Dendritic cell-based**							
**Species**	**Number and baseline characteristics**	**Loading strategy**	**Antigen**	**Results**			**References**
Human	6 therapy- naïeve	Pulsing	Recombinant HIV-1 MN gp160 or synthetic peptides corresponding to HLA-A2- restricted cytotoxic epitopes of envelope, Gag, and Pol proteins	Well tolerated and no effect on viral load. HIV specific responses were enhanced.			[100]
Human	4 HAART	Pulsing	Seven CTL peptides with HLA-A*2402 restriction	Well tolerated, discontinuation of HAART after vaccination failed to lower viral set points. CD8 T cell responses induced in 2 out of 4 patients.			[101]
Pigtail macaque	36 HAART	Pulsing of whole blood	Gag proteins or peptides spanning all 9 SIV proteins	SIV-specific CD4 and CD8T cell responses during antiretroviral cover and off treatment. Virus levels were 10-fold lower in immunized animals for 1 year.			[102]
Chinese rhesus macaques	HAART	Pulsing	AT-2 inactivated virus	Effective and durable SIV-specific cellular and humoral immunity is elicited. At week 34 of the study: 50-fold decrease of SIV DNA and a 1,000-fold decrease of SIV RNA.			[103]
Human	18 therapy naieve	Pulsing	AT-2-inactivated virus	Plasma viral load levels were decreased by 80% (median) over the first 112 days following immunization. The suppression of viral load was positively correlated with HIV-1-specific interleukin-2 or IFN-γ expressing CD4 T cells and with HIV-1 gag-specific perforin-expressing CD8 effector cells.			[104]
Human	12 HAART	Pulsing	Heat-inactivated virus	Safe and well tolerated. Partial viral control 24 weeks after ATI .			[105]
Human	24 therapy- naïeve	Pulsing	Heat-inactivated virus	Feasible, safe and well tolerated. Modest decrease in viral load 24 weeks after first vaccination compared to controls.			[106]
Human	29 HAART	Live virus	ALVACvcp1452	Viral load rebounded in both groups no differences in HIV-specific immune responses.			[107]
			ALVACvcp1452 + KLH				
			KLH				
Human	9 HAART	Electroporation	Autologous mRNA encoding Gag, Vpr, Rev and Nef	Mild adverse events. Full or partial HIV-specific immune responses in 7/9 subjects.			[108]
Human	9 HAART	Electroporation	Autologous mRNA encoding Gag, Vpr, Rev and Nef	Partial viral control .			[109]
Human	17 HAART	Electroporation	mRNA encoding tat rev nef	Vaccine was safe, 69 weeks after STI 6/17 patients remains off therapy.			[110]
Human	6 HAART	Electroporation	mRNA encoding gag and mRNA encoding tat rev nef	Vaccine was safe. HIV-specific responses against Gag were broader, higher and polyfunctional after vaccination. CD8 T-cells could inhibit superinfection of CD4 T-cells.			[111]

#### Remune®

Remune® was derived from a Congolese clade A/G HIV-1, which was gp120 depleted, chemically inactivated, irradiated, and emulsified with incomplete Freund's adjuvant [112]. The first uncontrolled clinical trials all showed that Remune® induced HIV-specific T cell responses [78]. In an open Remune® trial in chronically infected subjects, with suboptimal drug treatment, a significant delay in viral rebound after treatment interruption (TI) was observed [78]. Another open trial in asymptomatic non-treated HIV-infected individuals reported higher CD4 and CD8 T-cell counts and stable VL [79]. However, these beneficial effects were not observed in a randomized, double-blind, placebo-controlled study with patients, receiving full HAART during acute infection and vaccinated with either placebo, ALVAC (canarypox expressing env, gag, pol and nef - see below) or with ALVAC + Remune®. After analytical treatment interruption (ATI), there was no difference in viral rebound between the three arms [80].

#### Plasmid DNA vaccination

DNA-based vaccines have the conceptual advantage over other vaccines in that they can express both viral antigens and “molecular adjuvants”, such as cytokines or costimulatory molecules. Moreover, they can be administered repeatedly without inducing anti-vector immunity. DNA vaccines have successfully been used prophylactically in various infectious models in rodents, but they are less efficient in primates. Nevertheless, repeated intramuscular (im) immunization with carefully designed optimized SIVmac239 *gag* and *env* plasmids by Pavlakis’ group resulted in strong cellular and humoral responses in naïve Indian rhesus macaques and a significant sustained reduction of VL upon mucosal infection with the related SIVmac251 [81]. This DNA vaccine was further improved by adding a plasmid encoding Nef-Tat-Vif (NTV) fusion protein and applying *in vivo* electroporation [82].

The same group of researchers next used this strategy in a therapeutic setting, comparing HAART alone or HAART + 3 DNA vaccinations in chronically SIV-infected animals. A significant increase in cellular responses was noted in the vaccinated animals. Upon ATI, the HAART-only treated animals showed a full-blown viral rebound and gradually progressed to AIDS, whereas only a limited viral rebound was observed in the vaccinated animals and a 10 times lower VL was sustained for 3 years [113]. At that time, the combination of HAART and therapeutic DNA vaccine was repeated in some of the originally vaccinated animals, using an improved strategy with a DNA construct, containing the 5 SIV genes + IL-15 + IL-15 receptor α genes, and using *in vivo* electroporation. This repeated vaccination resulted in sustained polyfunctional central memory and effector memory CD4 and CD8 T cell responses and, importantly, an additional 1 log reduction in VL load after ATI [114]. These studies therefore provide proof-of-principle for plasmid DNA as a therapeutic vaccine.

However, in HAART-treated HIV-infected subjects the evidence for success of DNA vaccination has not yet been delivered. Repeated im injections of a DNA vaccine, based on CTL epitopes from multiple HIV-1 gene products elicited only weak T cell responses in HAART treated chronic patients [83]. The VRC-HIVDNA 009-00-VP, consisting of 4 plasmids encoding a subtype B Gag-Pol-Nef fusion protein and modified multiclade envelope constructs, was tested in a double blind placebo controlled study on patients treated with HAART during the acute/early phase. Even four im injections remained poorly immunogenic, and there was no effect on viral rebound after ATI [84]. A similar study in chronically treated patients, using DNA plasmids containing genes of several HIV-1 subtypes, apparently resulted in broader and higher HIV-specific T cell responses, but again there was no favorable effect on the viral rebound after ATI [85]. The DermaVir concept, recently reviewed by Lori, features a single plasmid DNA expressing 15 HIV antigens, a nano-particular formulation and a dendritic cell (DC) targeting topical (skin patch) administration. An impressive amount of safety and immunogenicity studies has been accumulated, but no statistically convincing data on VL reduction have been presented yet [115,116].

In a preventive setting, co-delivery of the genetic information to produce IFN-γ, IL-12, IL-15 or IL-18 has been shown to enhance T cell responses to SIV or HIV DNA constructs in non-infected macaques [117]. A favorable immune enhancement effect of co-delivered IL-12 or IL-15 genetic information was confirmed in a therapeutic setting in SIV-infected macaques [118] as well as in HIV-infected chimpanzees [119]. A strong synergistic effect between IL-12 and *in vivo* electroporation was also observed in preventive DNA vaccination in macaques [120]. In a human preventive setting, however, co-administration of IL-12 and/or IL-15 plasmids im failed to enhance T cell responses to HIV-1 *gag* DNA vaccine [121]. *In vivo* electroporation might be a viable option in humans, as two recent studies in healthy volunteers confirm the afore mentioned macaque data in that the T cell responses to intradermally (id) applied plasmid HIV DNA, followed by *in vivo* electroporation, are much stronger and more polyfunctional than those induced by regular im injection without electroporation [122,123].

#### Viral vectors expressing HIV antigens

##### Canarypox vector (ALVAC)

ALVAC is a recombinant canarypox vaccine, expressing full length *env* and *gag* as well as portions of *pol* and *nef.* The vCP1521 variant, expressing subtype E *gp120* and *gp41*, *gag* and *protease* of subtype B, was used as a prime for AIDSVAX (VaxGen bivalent gp120 subtype B/E), the recombinant glycoprotein boost in the prophylactic RV144 “Thai” trial, which showed a 31.2% protective efficacy against HIV acquisition [124]. The mechanisms of this protection are still under active investigation.

ALVAC vCP1433 and vCP1452, based on subtype B LAI sequences have been used in several therapeutic settings with varying success. The open-label single arm ANRS 094 study used 4 monthly im ALVAC injections, showing safety and immunogenicity [86]. The ANRS 093 trial used 4 im injections of ALVAC and Lipo-6T, a mixture of HIV derived lipopeptides, followed by 3 sc cycles of IL-2 in chronic HIV patients under stable HAART and included a control group under HAART alone. After ATI, a larger proportion of subjects in the vaccine group managed to lower their viral setpoint (24% vs 5%), and this was correlated with an enhanced vaccine-induced CD4 T cell response [87]. A follow-up study in HAART patients (ACTG A5024) included 4 arms, comparing placebo with ALVAC-HIV alone, sc IL-2 + ALVAC placebo, and the combination ALVAC-HIV and IL-2. Viral rebound, assessed 12 weeks after ATI, was 0.5 logs lower in both ALVAC-HIV vaccinated groups, whereas IL-2 increased CD4 T counts but did not diminish VL [88]. These early studies suggest that ALVAC vaccination can partly prevent viral rebound in chronically infected HAART patients.

As mentioned, a double blind placebo controlled trial with ALVAC-HIV or ALVAC-HIV and Remune® in patients already treated in the acute phase (QUEST), confirmed induction of immune responses, but did not result in better virological control 24 weeks after interruption of HAART [80]. A very similar study in chronic patients (CTN173) confirmed that both vaccines were unable to lower the viral setpoint, but nevertheless tended to delay rebound and extend time to restart HAART, which was also marginally correlated with higher IFN-γ and IL-2 responses [89].

Finally, a rather disappointing result was obtained in the ORVACS study, where 4 and 3 injections of ALVAC vCP1452 were compared with placebo in chronic HAART patients. The vaccines were immunogenic, but both vaccinated groups showed a higher viral rebound and had to resume treatment more rapidly than the placebo group [90]. A lower CD4 nadir and a higher vaccine-induced HIV-specific CD4 T cell cytokine response in Enzyme Linked Immunosorbent Spot Assay (ELISPOT) were predictive of this adverse outcome [125]. This result is somehow reminiscent of the prophylactic STEP trail, where vaccine-induced CD4 T cell activation has been invoked to explain apparently enhanced susceptibility to HIV infection [126].

Clearly, these various ALVAC trials provided rather contradictory results. Nevertheless, they indicate that baseline characteristics of patients (e.g. pre-treatment VL or CD4 T cell nadir) need to be carefully matched, and they confirm the notion that vaccine-induced immune activation can have both beneficial and adverse effects: whereas HIV-specific T cell activation is required to control viral rebound, immune stimulation can also increase the susceptibility of CD4+ target cells to productive infection.

##### Fowl pox

An Australian group compared two recombinant fowl pox vectors: one expressing *gag/pol* from subtype B only (a so-called partial construct or PC), while the second co-expressed human IFN-γ (full construct or FC), in addition to a placebo (diluent only). Thirty five fully HAART-treated subjects were randomized to the 3 regimens (12 placebo −11 PC −12 FC) and received 3 im injections (week 0, 4 and 12), and were then followed up for 52 weeks under HAART [91]. A subset (7 placebo; 8 PC and 10 FC subjected) underwent ATI for 20 weeks thereafter [92]. The most remarkable observation was a significantly lower mean viral rebound in the FC patients (+ 0.96 log), as compared to placebo (+ 1.80 log) and PC (+ 1.78 log). The T cell responses during the vaccination period were very weak. During ATI the IFN-γ ELISPOT increased in all three groups, but the changes were higher in the placebo as compared to the vaccinated patients. In a post hoc analysis, however, it was found that IgG2 antibodies to HIV p24 were present at 52 weeks (time of ATI start) in 5/9 FC patients and not in the PC or placebo patients. The presence of these antibodies was associated with lower viral rebound [127]. Clearly, the addition of IFN-γ to the vaccine seemed to be beneficial, and IgG2 antibodies were implicated in this protective effect.

##### Modified Vaccinia Ankara (MVA)

Several constructs expressing various HIV or SIV genes in replication-deficient poxvirus have been used as prophylactic vaccines in macaques, preceded by plasmid DNA priming with the same antigens. Partial, but sustained, protection has repeatedly been shown against homologous challenges: in most cases infection could not be prevented, but VL was lower and disease progression delayed or halted [128-130]. Whereas correlations with neutralizing antibodies as well as CD4 and CD8 T cell responses were noted in some cases, a crucial role for CD8 T cells in this protection was strongly suggested by a CD8 depletion approach in the study of Amara [131].

A few studies addressed the potential therapeutic effect of MVA expressing SIV antigens in infected macaques under antiretroviral treatment. A small trial using MVA *gag-pol*/MVA *env* or MVA t*at-ref-nef* in SIV-infected animals, treated with the NRTI PMPA, showed a tendency towards lower viral rebound after ATI in the vaccinated groups [93]. A regimen with combined Adenovirus constructs (see below) and MVA expressing *gag* and *env* in infected and treated macaques provided a more sustained reduction in VL and increase of CD4 T cell counts, correlated with increased anti-SIV cell-mediated and humoral responses [94].

In HIV-infected subjects under HAART, *nef*-expressing MVA was shown to induce CD8 and CD4 T cell responses in some patients and there was a relatively lower viral rebound after treatment interruption [95]. An elegant series of studies was carried out in 16 chronically infected patients under HAART, using an MVA construct expressing consensus clade A p24/p17 and multiple CD8 T cell epitopes. The authors showed that both CD4 and CD8 T cell responses were amplified and broadened, and that CD8 T cells acquired the capacity to inhibit HIV-1 replication *in vitro*[96,97]. Especially the latter characteristic seems most important for potential therapeutic effect.

Other groups have also developed promising HIV MVA constructs that elicited polyfunctional CD4 and CD8 T cell responses in either healthy or HIV infected subjects [132-134], and more formal testing in therapeutic trials is presumably ongoing.

##### Adenovirus constructs

Two phase IIb studies, STEP and Phambili, evaluated the prophylactic effect of the replication- defective recombinant adenovirus type 5 (rAd5) MRK *gag/pol/nef* vaccines in healthy volunteers. Clearly, neither trial showed a decrease in HIV acquisition, nor decreased early plasma VL in vaccinees, who were infected. A post-hoc analysis showed even an increased risk of infection in uncircumcised vaccinated men, who were already Ad5 seropositive before vaccination [135]. This deleterious effect was tentatively explained by the observation that Ad5 could induce expansion of memory CD4 T cells with a mucosal homing phenotype, which are readily susceptible to HIV-1. Several studies provided evidence that the untoward effect of pre-existing Adeno immunity by natural infection could have a negative impact on immune responses against HIV and could increase susceptibility towards HIV infection, even if rare Adeno serotypes were being used [126,136,137].

Human replication deficient rAd5 and rAd35 vectors, encoding SIV *gag, env* and *nef* (with or without IL-15 encoding cassettes), were used in chronically SIVmac251 infected and properly treated macaques, followed by ATI. HIV specific T cells were increased but viral rebound was not influenced [98].

The AIDS Clinical Trial Group (ACTG) protocol A5197 was a randomized placebo controlled trial to test rAd5 expressing *gag* in chronically infected patients under stable HAART with 77 persons in the vaccinated and 37 in the placebo group. Interestingly, 16 weeks after ATI, the plasma VL was 0.5 logs lower in the vaccinated group [99].

Though the therapeutic trial in SIV-infected macaques was not promising, the rather positive result of the ACTG trial in chronic HAART patients raises some hope. There are several recent studies in seronegative controls indicating that a prime-boost regimen with HIV *env* and *gag/pol* DNA/Ad5 may enhance antibody titers and T cell responses. Importantly, the T cells showed not only increased poly-functionality, but also a significant HIV-suppressive effect towards several HIV strains, including transmitted/founder viruses *in vitro*[138,139]. Another possible way forward is the subsequent vaccination with rAD5 and rMVA, with synergistic activities on effector memory and central memory CD8 T cells [140].

#### Dendritic cell based immunotherapy

A number of DC-based therapeutic trials have recently been completed in animals and humans. DC have the capacity to process proteins through both MHC class I and class II pathways for stimulation of CD8 or CD4 T cells respectively. Antigen can be provided to DC in many formats: as peptides, whole proteins or apoptotic cells, and also in a “genetic” format by transfecting DC with antigen-encoding viral vectors in DNA or mRNA format.

a) *Antigenic peptides and recombinant proteins*

The use of antigenic peptides is an efficient loading strategy, but DC can also be pulsed with recombinant HIV proteins [24]. In principle, “exogenous” protein should preferentially induce MHC (HLA)-class II restricted CD4 T cells, whereas peptides could stimulate both CD4 and CD8 T cells, according to their class I or II binding, depending on their length and composition. The first small trial in humans was performed by Kundu and showed that the administration of HIV peptides or protein-pulsed autologous DC was well-tolerated and could enhance the immune response to HIV in therapy-naïve patients with normal CD4 T cell counts [100]. In a second trial, 6 doses of synthetic HIV-1 peptide-pulsed autologous DC were administered to 4 HAART-treated, HLA-A2402 individuals who underwent ATI: no significant changes in VL or CD4 T cells were observed during ATI [101]. Finally, De Rose et al. performed an intriguing trial in pigtail macaques, where blood was exposed *ex vivo* to overlapping SIV peptides or medium for 1 hour and re-injected. This procedure was repeated 7 times. SIV specific CD4 and CD8 T cell responses were induced; and, remarkably, virus levels were approximately 10-fold lower for 1 year in immunized animals as compared to medium controls [102]. A basic problem with peptides and proteins, however, is that it is difficult to cover HIV variability and HLA polymorphisms at an affordable cost.

b) *Whole inactivated virus*

Whole inactivated HIV-1 particles have successfully been used in DC vaccination of mice, monkeys and humans. Two prophylactic trials were performed in severe combined immunodeficient (SCID) mice, reconstituted with human PBMC. Yoshida and colleagues used IL-4 DC (i.e. monocytes differentiated into DC in the presence of IL-4 and GM-CSF and matured with IFN-α), whereas Lapenta and colleagues used IFN-DC (i.e. monocytes differentiated into DC in the presence of IFN-α and IL-4). In both trials, autologous DC were pulsed with aldrithiol (AT)-2-inactivated R5 subtype B viruses: HIV-1_SF-162_ by Lapenta [141] and HIV-1_JR-CSF_ by Yoshida [142]. After vaccination with the pulsed DC, HIV-1 specific CD4 and CD8 T cells were generated *in vivo*. Moreover, upon infection with homologous virus, there was also evidence for partial protection.

Around the same time, Lu et al. published their paper on therapeutic vaccination in Chinese rhesus macaques [103]. They used AT-2-inactivated SIVmac251-pulsed IL-4 DC, matured with the classical “Jonuleit” cytokine cocktail, consisting of IL-1β, tumor necrosis factor-α, IL-6 and Prostaglandin E_2_[143]. All animals displayed a significant decrease in viral load 10 days after vaccination and an increased CD4 T cell count. Clearly enhanced SIV-specific cellular immunity was also observed.

The same authors vaccinated 18 untreated chronically HIV-infected patients, using IL-4 DC pulsed with autologous AT-2-inactivated virus. This resulted in an effective HIV-1-specific T cell response with sustained viral suppression of over 90% in 8 of 18 subjects [104]. Based on statistical correlations, robust virus-specific CD4 T helper cells were required to induce and maintain virus-specific CD8 T effector cells for virus containment.

In another clinical trial, Garcia used heat-inactivated autologous virus to pulse IL-4 DC: in 12 HIV-1-infected persons under HAART, partial viral control could be achieved 24 weeks after ATI [105]. In contrast to the observations of Lu et al., the HIV-specific cellular immune response was weak and transient in Garcia’s study. The latter author also performed a double blind clinical trial on untreated HIV-1 infected patients with IL4 DC pulsed with heat-inactivated virus [106]. VL in the active group was maintained at a lower level as compared to the placebo group at week 48. However, this result was inversely correlated with HIV-1 specific immune responses.

Clearly, although Lu’s and Garcia’s vaccination strategies both yielded a positive effect on VL, the association between VL and T cell responses was different. Whether this discrepancy is due to the different inactivation procedure (AT2 vs. heat inactivation) or other factors remains to be investigated.

Unfortunately, DC loading with inactivated virus is difficult to standardize due to numerous variables: type and activation state of infected cells, method of virus inactivation, and the amount of antigen in the preparation. Moreover, it requires meticulous quality control testing on the inactivation procedure in order to eliminate any biological risk of infection.

c) *Live viral vectors*

As already discussed, direct vaccination with Canarypox virus vectors carrying HIV-1 genes (ALVAC) yielded discordant results in several studies. Recently a phase I/II clinical trial was performed, comparing direct injection of ALVAC vCP1452 and keyhole limpet hemocyanin (KLH) as adjuvant with injection of autologous IL-4 DC infected *ex vivo* with ALVAC vCP1452 and treated with KLH. After three injections, subjects underwent a minimum of a 12-week ATI. Viral load rebounded in both groups and there was also no difference in HIV specific responses [107].

d) *mRNA and DNA*

Loading HIV antigens encoded by nucleic acid, either cDNA or mRNA, is easier to standardize, it does not carry infectious risk, and hence seems straightforward for clinical applications. In our hands, transfection of IL-4 DC with cDNA is less attractive than mRNA, as cDNA electroporation resulted in more cell death and expression levels were lower, probably due to the more stringent transformation conditions required to penetrate into the nucleus. Transfection with mRNA encoding antigens requires cytoplasmic penetration only and was very efficient for loading DC and subsequent stimulation of HIV-specific T-cells [144].

Several clinical trials have already been performed based on DC electroporated with mRNA encoding HIV-1 proteins. In AGS-004, nine HAART-treated individuals were vaccinated with autologous IL-4 DC electroporated with mRNA encoding CD40L and autologous HIV antigens Gag, Vpr, Rev and Nef. Patients received monthly injections in combination with HAART [108]. Seven out of nine patients showed proliferative CD8 T cell responses. This vaccine was further evaluated in a phase II study, resulting in partial viral control [109]. Another recent clinical trial was performed by Allard et al. In this study, 17 HIV infected individuals on HAART received 4 vaccinations with 4 week intervals of autologous IL-4 DC electroporated with mRNA encoding a subtype B TatRevNef fusion protein. Four weeks after the last vaccination, treatment was interrupted [110]. Six out of 17 patients remained off therapy 69 weeks after ATI. However this clinical result was not better than a historic control group undergoing STI, despite the induction and enhancement of CD4 and CD8 T cell responses specific for the vaccine [110]. Around the same time our group also performed a phase I/II clinical trial: 6 HIV-infected individuals who received stable HAART were included. Individuals were vaccinated with autologous IL-4 DC electroporated with mRNA encoding the same TatRevNef fusion protein as in Allard’s study and with IL-4 DC expressing subtype B Gag mRNA. After vaccination, HIV-specific responses to Gag were increased in magnitude, breadth and proliferative capacity. Although no virological parameters could be measured because patients did not undergo ATI, we showed that the CD8 T cells from the vaccines could inhibit superinfection of autologous CD4 T cells with vaccine related IIIB virus *in vitro*[111].

Overall, 210 patients (60 therapy naïve and 150 on HAART) have been recruited in clinical trials with DC based vaccines. The safety profile has been excellent and DC therapy clearly elicits HIV-1 specific immunological responses, but only four of these studies reported virological responses to immunization [145].

e) *In vivo targeting of dendritic cells*

Current immunotherapeutic strategies involve the *ex vivo* manipulation of autologous DC. This vaccination procedure is labor-intensive, logistically complicated, expensive and not useful in developing countries. A possible solution is direct *in vivo* delivery of suitable antigens and co-stimuli to resident DC. A first attempt in animal models was to apply protein antigens in complex with antibodies to DC-specific membrane molecules such as DC-SIGN or DEC-205 [146,147]. A future strategy could be the delivery of biodegradable nanoparticles, which will be taken up by endocytic DC *in vivo*, such as the DermaVir patches [115].

## Conclusions

Early attempts of immunotherapy, during the pre-HAART era, included non-specific (e.g. IFN and IL-2) and HIV-specific (e.g. Remune) immune interventions in patients who were either untreated or received mono- or dual drug therapy. In some cases positive effects have been reported, but these trials were small and usually not well controlled. During the last 10–15 years, the concept of immunotherapy as a supplement to full HAART has been further developed, with the ultimate aim to reduce or even replace drug therapy.

Amongst the non-antigen-specific immune approaches, systemic IL-2 has been exhaustively investigated and was ultimately shown not to provide clinical benefit in addition to HAART. It remains to be seen whether systemic use of other common γ chain cytokines, such as IL-7 or IL-21, could be useful for particular indications (e.g. to counteract lymphopenia). The newer “non-specific” approach is to block negative regulatory pathways, such as PD-1 or CTLA-4 signaling, both over-expressed during HIV infection. Experimental treatment in SIV-infected macaques indicated that PD-1 blocking helped to control viremia and to reduce immune activation. In contrast, CTLA-4 blocking resulted in increased viremia, most probably as a result of an unfavorable balance between too much non-specific and insufficient specific immune activation.

The obvious goal of therapeutic vaccination is to stimulate effective HIV-specific T cell responses, primarily cytolytic/virus suppressive CD8 T cells with supporting CD4 T cell help, while avoiding increased HIV-susceptibility of target cells. To that end, protein or particulate antigen is conceptually not the best option, since it primarily results in CD4 T and B cell activation: antibodies are considered ineffective and CD8 T cells seem essential in immune protection post infection.

Repeated im vaccination with plasmid DNA encoding various HIV antigens, along with cytokines, was successful in lowering VL in macaques, but this has not yet been confirmed in humans, despite repeated attempts. However, the immunogenicity of DNA vaccination is being improved by innovative constructs and enhanced delivery systems [148]. Especially for applications in less sophisticated environments, DNA vaccines have the advantage of being stable and to carrying no infectious risk, but because they are considered “genetic therapy”, the development of DNA vaccines is subjected to strict regulations [149].

Recombinant viral vectors have been applied rather extensively in therapeutic vaccination trials. In most cases poxviruses (e.g. canary pox, fowl pox and MVA) and occasionally Adenoviruses were used. A fair number of encouraging results were observed in infected/treated macaques and patients in terms of T cell responses, and in some cases also VL reduction after ATI was observed. However, the variable results, obtained with the most extensively used canarypox platform, ALVAC, illustrate that apparently similar trials can have quite discrepant outcomes. Again, this may be the result of a delicate balance between the beneficial enhancement of anti-HIV CD8 T cell immunity and the deleterious effect of non-specific immune activation, inducing increased cellular susceptibility to HIV infection.

DC-based therapy has been developed in a therapeutic context, with various antigenic formats and corresponding loading strategies. Remarkably positive results in terms of viral control have been obtained with autologous inactivated virus loaded DC. DC trials with safer and less cumbersome formats (e.g. RNA) are ongoing, but the first results suggest that there is a need for improvement and further simplification of this methodology.

Collectively, it seems that HIV immunotherapy, although still highly experimental, is a viable option to explore. Although some beneficial effects have been described in untreated individuals, most evidence today argues that more effective immune responses can be induced under HAART coverage. In most trials, potentially favorable T cell responses were induced, while effects on VL have at best been transient. This limited success should not come as a surprise, since in most cases antigens from one particular or a limited number of HIV strains were used as immunogen and even in those trials, where the “autologous” virus or sequences thereof were used, epitopes from which the immune system had already escaped may have been presented.

To overcome these restrictions, new options are available. One is to immunize with a set of the most conserved, subdominant epitopes, mainly in the structural core genes (i.e. Gag) in order to focus and “teach” T cells to effectively react against at least some determinants which the virus cannot possibly escape from without completely losing infectivity. Obviously, this strategy might require repeated immunizations with sufficiently large numbers of different stretches of antigens, in view of the requirement to accommodate the HLA restrictions and to allow T cell receptor “maturation” by gradual somatic mutations. One such approach was proposed by Letourneau et al. [150]. With these antigens, strong immune responses were already induced both in mice and macaques, using HIV conserved alphavirus replicon DNA and electroporation [151]. A similar approach, focusing on different epitopes was proposed more recently by C. Brander [152]. A second alternative is the so-called mosaic *gag* approach, where a large number of natural HIV sequences are included by *in silico* recombination (ftp://ftp-t10.lanl.gov/pub/btk/mozaïek) for maximum coverage of potential T cell epitopes of HIV-1 group M, including potential escape variations [153,154]. This approach has successfully been applied in macaques, using DNA + recombinant vaccinia boost [155-157]. Interestingly, this viral construct also induced broad responses in PBMC from HIV-1 B or C infected subjects [157].

The next important question to address is how to deliver this improved antigen *in vivo*. Clearly, genetic constructs, either plasmid DNA, mRNA or recombinant viruses are suitable. All three formats have advantages and drawbacks as explained. Our own preference goes to mRNA, as it is flexible and clean, can include a large number of antigens and variants, and does not carry any infection or insertional risk or risk for vector-related adverse immune reactions [25]. This safety advantage of mRNA-based vaccines as compared to plasmid DNA or viral vectors is reflected in their classification by the authorities (FDA in the USA and Paul Ehrlich Institute in Germany) as no gene therapy approaches. This relaxes the requirements concerning preclinical toxicology studies in animals [149].

Another feature of mRNA is, however, its biological instability (sensitivity to ubiquitous RNAses); and therefore it has mostly been used to load patient-derived DC *ex vivo*, a strategy which is not applicable at a larger scale. Recent evidence, however, indicates that mRNA can be chemically stabilized to be injected as “naked” RNA *in vivo* (e.g. intranodally) and/or formulated with either cationic lipids or positively charged proteins. The latter technologies have been refined over the years, and are now ready for clinical application [158-160].

In addition to the antigen and its format or formulation, further signals may be required to optimize the immune responses. Especially if the format is DNA or RNA, a clearly defined genetic adjuvant is most attractive, as it will restrict the costimulatory effect to the site where the immune response is elicited. Th1-skewing (IL-12, IFN-γ), common γ chain or cytokines, TNF ligand members or Toll-like receptor ligands have been used with some success [28]. From our review two new candidates emerge: IL-21 [45] and PD-1 blocking agents [161].

The desired clinical outcome of a therapeutic vaccine is to keep the VL under control and restore CD4 T cell counts through CD8 T cell-mediated suppression of viral re-emergence. However, there are no easy correlates for this protective effect while the patient is still on HAART. A straightforward ELISPOT using a standard subtype B set of peptides provides a first indication of immunogenicity, but it does not represent a true correlate for protection. Expanding the breadth of the T cell responses by testing many peptide variants, “potential T cell epitopes” (PTE) [162], increasing avidity by testing low peptide concentrations or improving the “quality” in terms of lytic capacity of CD8 T cells or poly-functionality as well as expansion of HIV-specific effector-memory or central memory CD4 and CD8 T cells have all been proposed as useful [6,8], but these tests remain somewhat indirect correlates and require rather delicate and cumbersome experimental approaches. As mentioned, several authors, including ourselves, have proposed to measure the vaccine-induced capacity of the patients’ CD8 T cells to suppress various HIV isolates *in vitro*, as this function has repeatedly been associated with the “elite controller” status [18-20,111]. Obviously, this type of functional test is also difficult to standardize, but it has the conceptual advantage of being a direct correlate of the desired protective effect.

Another important parameter to consider is whether immunotherapy or any other intervention aiming at a “functional cure” could reduce the size of the latent reservoir. Several assays have been proposed to measure residual replication-competent virus in purified CD4 T cells, based on (enhanced) viral culture as well as on an array of PCR-based methods quantifying intracellular viral mRNA (unspliced or multiple spliced) or proviral DNA. With regard to the latter, it is important to determine integrated versus non-integrated proviral DNA as well as the precise cell subset of the reservoir (activated versus resting T cells, central-memory versus terminally differentiated, macrophages, dendritic cells etc.). Moreover, the measurement should not be limited to blood, but also consider reservoirs in lymphoid tissues (peripheral or gastro-intestinal-associated lymph nodes) as well as “sanctuary sites” (such as the CNS or the genital tract). While the conceptual importance of these measurements is evident, these assays are presently not standardized and it remains to be determined which ones are most predictive of what we really want to know: will our intervention be able to mitigate or abrogate viral rebound after subsequent interruption of HAART [1,30]?

Since there is no definite *ex vivo/in vitro* correlate of protection and since the SIV macaque model does not necessarily predict effects in human trials, one needs to proceed to a human trial with analytical treatment interruption to assess the ultimate clinical effect. In view of the observed variability in trial outcome, there is a clear requirement for a carefully matched and sufficiently large placebo group. Treatment interruption, even when guided by CD4 T cell counts and carefully designed to avoid emergence of drug resistance, is being considered as harmful in the long run since the SMART trial [29]. To address this problem, Routy recently reanalyzed the SMART data for the effects of a limited duration of treatment interruption (e.g. 16 weeks) in patients on stable HIV-suppressive HAART with CD4 T counts > 400/μL, a CD4 T cell nadir of > 200/μL and without concomitant diseases. He concluded that ATI under those conditions is safe and thus acceptable in the context of a well monitored therapeutic trial with timely resumption of HAART in case of rebound and/or CD4 T cell drop [163].

## Abbreviations

AIDS: Acquired immune deficiency syndrome; ANRS: Agence Nationale de Recherche sur le SIDA; APC: Antigen-presenting cell; AT-2: Aldithriol; ATI: Analytical treatment interruption; CTL: Cytolytic T cell; CTLA-4: Cytolytic T lymphocyte antigen-4; DC: Dendritic cell; EC: Elite controllers; HAART: Highly active antiretroviral therapy; HIV: Human immune deficiency virus; HSC: Hematopoietic stem cells; Id: Intradermal; IFN: Interferon; IL: Interleukin; Im: Intramuscular; ISG: Interferon-stimulated genes; Iv: Intravenous; MVA: Modified vaccinia Ankara; NK: Natural killer; NRTI: Nucleoside reverse transcriptase inhibitor; PBMC: Peripheral blood mononuclear cells; PD-1: Programmed death receptor-1; PTC: Post-treatment control; Sc: Subcutaneous; SCID: Severe combined immune deficiency; SIV: Simian immune deficiency virus; STI: Structured treatment interruption; Th: T helper; TI: Treatment interruption; Treg: Regulatory T cell; VL: Viral load; W: Week.

## Competing interests

The authors have no competing interests to declare.

## Authors’ contributions

GVH an EVG conceived and wrote the review together. Both authors read and approved the final manuscript.
